# Supplementary material for: A robust, sensitive phylogenetic method enables gene-level metagenomic analyses
Source: bioRxiv. 2026 Jul 15:2026.07.15.738679. Preprint. [Version 1] doi: 10.64898/2026.07.15.738679 (PMC13404960; doi:10.64898/2026.07.15.738679)
Supplement: 1 [file NIHPP2026.07.15.738679v1-supplement-1.pdf]

bioRxiv preprint doi: https://doi.org/10.64898/2026.07.15.738679; this version posted July 15, 2026. The copyright holder for this preprint (which was not certified by peer review) is the author/funder, who has granted bioRxiv a license to display the preprint in perpetuity. It is made available under a CC-BY 4.0 International license.

Supplemental Figure and Table Legends

Supplemental Figure 1: Full results of simulations from Figure 4 (excluding uncorrected linear regression).

Supplemental Figure 2: Full results of simulations from Figure 4 (including uncorrected linear regression).

Supplemental Figure 3: Two top hits specific to the uncorrected linear model applied to *Lachnospiraceae* in liver cirrhosis, EF-Tu and DNA polymerase III.

Supplementary Table 1: Positive gene associations with human gut *Lachnospiraceae* in cirrhosis that were significant ($q<0.05$) via robust permutration (“perm”), the linear model (“lin”), and/or POMS (“poms”).

Supplementary Table 2: Genome neighborhood network analysis for C0FN14 showing overrepresented Pfam domain architectures. Domain architectures that match a protein discussed in the paper are noted in the "annotation" column.
